# Comparison of variable selection procedures and investigation of the role of shrinkage in linear regression-protocol of a simulation study in low-dimensional data

**DOI:** 10.1371/journal.pone.0271240

**Published:** 2022-10-03

**Authors:** Edwin Kipruto, Willi Sauerbrei

**Affiliations:** Institute of Medical Biometry and Statistics, Faculty of Medicine and Medical Center - University of Freiburg, Freiburg, Germany; Institute for Quality and Efficienty in Health Care (IQWiG), GERMANY

## Abstract

In low-dimensional data and within the framework of a classical linear regression model, we intend to compare variable selection methods and investigate the role of shrinkage of regression estimates in a simulation study. Our primary aim is to build descriptive models that capture the data structure parsimoniously, while our secondary aim is to derive a prediction model. Simulation studies are an important tool in statistical methodology research if they are well designed, executed, and reported. However, bias in favor of an “own” preferred method is prevalent in most simulation studies in which a new method is proposed and compared with existing methods. To overcome such bias, neutral comparison studies, which disregard the superiority or inferiority of a particular method, have been proposed. In this paper, we designed a simulation study with key principles of neutral comparison studies in mind, though certain unintentional biases cannot be ruled out. To improve the design and reporting of a simulation study, we followed the recently proposed ADEMP structure, which entails defining the aims (A), data-generating mechanisms (D), estimand/target of analysis (E), methods (M), and performance measures (P). To ensure the reproducibility of results, we published the protocol before conducting the study. In addition, we presented earlier versions of the design to several experts whose feedback influenced certain aspects of the design. We will compare popular penalized regression methods (lasso, adaptive lasso, relaxed lasso, and nonnegative garrote) that combine variable selection and shrinkage with classical variable selection methods (best subset selection and backward elimination) with and without post-estimation shrinkage of parameter estimates.

## 1. Introduction

Over the last decade, considerable attention has focused on penalized regression models, and several methods have been proposed owing to their computational efficiency in variable selection with many potential candidate covariates [1]. There is a consensus that the choice of a method depends on the aim of the study. This simulation study focuses on descriptive modeling that aims to capture the association between response and covariate variables. As such, simple models, which are more interpretable and transferable than complex models containing a large number of variables are preferred [2]. In an overview of methods for selection of variables and functional forms for continuous variables, seven important topics that warrant further research, ideally by well-designed and analyzed simulation studies, were highlighted [3]. Here we will concentrate on (i) investigation and comparison of properties of variable selection strategies and (ii) the role of shrinkage in the correction of selection bias introduced by data-dependent modeling.

Following the general objectives of the STRengthening Analytical Thinking for Observational Studies (STRATOS) initiative (https://stratos-initiative.org/), which aims to derive evidence supported guidance for the design and analysis of relevant issues in observational studies [4], we will compare variable selection procedures that have been in existence for many years. In penalized likelihood procedures that combine variable selection and shrinkage, we will evaluate the nonnegative garrote (NNG) [5], lasso [6], adaptive lasso [7], and relaxed lasso [8]. In addition, classical variable selection strategies, i.e., best subset selection and backward elimination, will be considered, and since their regression estimates are not shrunken, we will subject them to shrinkage obtained from post-selection shrinkage methods [5, 9, 10] and compare the results with penalized likelihood methods. It is important to note that the NNG is among the first proposed methods that combine variable selection and shrinkage. Therefore, the new proposal for suitable initial estimates makes its application possible even in highly correlated (HCD) and high-dimensional data (HDD) [11]. The lasso was proposed about a year after the NNG and is a special case of bridge regression introduced by Frank and Friedman [12]. The lasso is the most popular regularized method, probably due to its application in HDD and the availability of fast algorithms for the estimation of its solution [13]. However, it’s well-known that the lasso has several weaknesses [7, 8], and various modifications like the adaptive lasso and relaxed lasso have been proposed to correct the weaknesses.

Simulation studies are an important tool in statistical methodology research, provided that they are well designed, executed, and reported. They can be used to explore situations for which theoretical arguments are insufficient to determine whether the method of interest is valid in a specific real-life application or not or explore situations of interest that cannot be assessed using real data because the true values of the underlying parameters are needed. Additionally, simulation studies can highlight ideal and problematic scenarios for specific methods [14, 15]. Morris and co-authors [16] reviewed 100 simulation studies published in Statistics in Medicine in 2015 and found that simulation studies tend to be poorly reported and identified several areas for improvement. They described and advocated the ADEMP structure. This structured approach continues to gain more acceptance among researchers and we used it to describe the protocol of our simulation study. We intend to conduct the simulation study in low-dimensional data in the framework of a linear regression model with normal errors, but methods can be used more generally in the context of generalized linear models and censored survival data.

Bias in favor of a preferred method is probably more prevalent in simulation studies in which a new method is proposed and compared with existing methods. In this case, a simulation study is often used to demonstrate the benefits (and rarely the weaknesses) of a new method over existing methods. The danger is that the researchers are likely to create settings in which their methods perform well [14]. This is not good scientific practice, which is unfortunately contributed by many factors. For instance, reviewers might easily reject a new method if the authors highlight several limitations, while the pressure to publish for career reasons can also lead to poor research [17].

To overcome this problem, neutral comparison studies and good reporting of simulation results have been proposed. Neutral comparison studies do not aim to demonstrate the superiority or inferiority of a particular method but provide insight into the properties of methods [18, 19]. An ideal form of neutral comparison studies requires the involvement of a group of researchers who are well-versed with all methods of interest and do not have an interest in the success of any of the methods [19]. That is an extremely difficult task, but we aim to conduct a simulation study with key principles of neutral comparison studies in mind, though certain unintentional biases can never be ruled out. To reduce biases caused by the design, the protocol was sent to some members of topic group 2 (Georg Heinze) and the simulation panel (Anne-Laure Boulesteix and Tim Morris) of the STRATOS initiative for comments or suggestions on all aspects of the design. As proposed by Morris and co-authors, we decided to increase the transparency of our work by publishing this simulation protocol.

The paper is organized as follows: Section 2 provides an introduction to the simulation design, followed by five subsections ordered according to the ADEMP structure. Subsection 2.2 describes the aims of the simulation study, while subsection 2.3 describes the data-generating mechanisms, such as the correlation structure and sample size, used in detail. Subsection 2.4 briefly describes the variable selection methods, while subsection 2.5 describes the performance measures used to compare several approaches. Final remarks are given in section 3, while the software implementation is relegated to the Appendix, which also contains a detailed description of the methods.

## 2. Simulation design–improvement through the ADEMP structure

Simulation studies are important tools for assessing the properties of variable selection procedures and comparing alternative methods. Borrowing information from published simulation studies with related investigations is important to gain insight into the weaknesses and strengths of designs. In the present study, we incorporated some information from several published studies as discussed in section 1 of the S1 File. Besides borrowing some ideas from the published studies, we used other different settings such as explained variation (*R*^*2*^), sample size (n), correlation structure (C), and different settings of regression coefficients (β) for a broader perspective. We will consider 15 covariates, of which 7 have effects. Some of the investigations will be repeated with 15 additional uncorrelated noise variables. Following the ADEMP structure, we have summarized relevant issues in Table 1 and provided details in five subsections 2.2 to 2.6.

**Table 1:** Summary of the simulation design following the ADEMP structure.

**Aims** (section 2.2)	To compare variable selection methods using different tuning parameters (CV, AIC and BIC) or initial estimates in terms of model selection and prediction. To assess the usefulness of post-estimation shrinkage in the prediction of classical variable selection methods and compare the results with penalized methods. To compare the amount of shrinkage of regression coefficients of penalized and post-estimation shrinkage methods. To assess the performance of different methods in the presence of relatively many noise variables, in larger sample size, in relatively high correlation and when *R*^*2*^ approaches one.		
**Data generating mechanism** (section 2.3)	**Training/development dataset** (***X*** ~ ***N***_***p***_(**0**, **Σ**))where *p* = 15 and $\Sigma \in R^{p\times p}$; **Σ**_***ij***_ is equal to the correlation coefficient between covariate **x**_**i**_ and **x**_**j**_ ***Y* = *Xβ* + *ϵ*** where ***β*** ∈ (***β***_***A***_, ***β***_***B***_, ***β***_***C***_, ***β***_***D***_) and ***ϵ*** ~ ***N***(**0**, ***σ***^**2**^***I***_***n***_) **True regression coefficients (β) for 15 covariates** β_A_: 1.5, 0, 1, 0, 1, 0, 0.5, 0, 0.5, 0, 0.5, 0, -0.5, 0, 0 –From [20] β_B_: 1.5, 0, 0.5, 0, 0.5, 0, 0.25, 0, 0.25, 0, 0.25, 0, -0.25, 0, 0 –(modified β_A_) β_C_: 1,0, 1, 0, 1, 0, 1, 0, 1, 0, 1, 0, 1, 0, 0 –From [21] β_D_: 1, 1, 1, 1, 1, 1, 1, 0, 0, 0, 0, 0, 0, 0, 0 –From [21] **Correlation structure (*C*)** C1: Taken from [20]–low collinearity C2: Autoregressive structure with **Σ**_***ij***_ = 0.3^|i-j|^-low collinearity C3 Autoregressive structure with Σ_*ij*_ = 0.8|i-j|–moderate collinearity C4: Adapted from real study, body fat data–high collinearity ***R***^*2*^ **and sample size (*n*)** *R*^*2*^ = {0.20, 0.30, 0.50, 0.71}; *n* = {100, 400} **Number of scenarios (full factorial design) and simulation runs** *β* × *C* × *R*^2^ × *n* = 4 × 4 × 4 × 2 = 128 scenarios N = 2,000 simulation repetitions per scenario **Test dataset** New simulations with the same design as training dataset (***n***_***test***_ = **100, 000**) **Additional analysis** Additional analysis will be conducted with β_A_, C1, n = (400, 800) and a subset of *R*^*2*^ = {0.30, 0.50, 0.71, 0.8, 0.9}		
**Estimand/target of analysis** (section 2.4)	Selection status of each covariate and identification of the true model Shrinkage factors for each regression estimate Model prediction errors		
**Methods** (section 2.5)	**A. Variable selection methods**		
	Method	Tuning parameters	Initial estimates
	Lasso	10-fold CV, AIC & BIC	N/A
	Garrote	10-fold CV, AIC & BIC	OLS, ridge and lasso
	Alasso*	10-fold CV, AIC & BIC	OLS, ridge and lasso
	Rlasso*	10-fold CV, AIC & BIC	N/A
	Best subset	10-fold CV, AIC & BIC	N/A
	BE*	10-fold CV, AIC & BIC	N/A
	**B. Post-estimation shrinkage methods**: (i) Global [10], (ii) parameterwise [9] and (iii) Breiman’s method [5] Estimation method: (i) leave-one-out CV and (ii) 10-fold CV		
**Performance measures** (section 2.6)	Inclusion and exclusion of variables: FNR & FPR–subsection 2.6.1 classification of models: Probabilities–subsection 2.6.1 Prediction accuracy: Model error (ME)–subsection 2.6.2 Variability of ME within and between scenarios—section 5 in S1 File		

*Alasso, Rlasso and BE denote adaptive lasso, relaxed lasso and backward elimination; while FNR and FPR denote false negative rates and false positive rates, respectively.

### 2.1 Additional analyses

The main analysis will concentrate on a broad examination of 128 scenarios from a fully factorial design. We will extend the main simulation study with three additional cases: (i) we will take n = 400, β_A_, C1, *R*^*2*^∈{0.3,0.5,0.71} with 30 covariates (*i*.*e*., the 15 original covariates and 15 uncorrelated noise variables), in order to study the performance of different methods in the presence of additional noise variables, (ii) we will set n = 800 with the original 15 covariates, β_A,_ C1, and *R*^*2*^∈{0.3,0.5,0.71}, in order to study the performance of the methods in larger sample size and (iii) we will rerun the simulations with n = 400, β_A,_ C1, p = 15 original covariates while varying *R*^*2*^∈{0.3,0.5,0.8,0.9} to study the consistency of methods as *R*^*2*^ approaches one.

### 2.2 Aims

Our study aims to: (i) compare variable selection methods using different tuning parameters (Cross-validation(CV), Akaike Information Criterion (AIC) and Bayesian Information Criterion (BIC)) or initial estimates in terms of selecting a ‘nearly true’ model and prediction errors, (ii) assess the usefulness of post-estimation shrinkage factors in improving the prediction performance of best subset selection and backward elimination, and compare results with penalized regression methods, (iii) assess the performance of different selection methods in the presence of many noise variables, in larger sample size, in relatively high correlation and when *R*^*2*^ approaches one.

### 2.3 Data generating mechanisms

#### 2.3.1 Training dataset and explained variation

A matrix of continuous covariates $X\in \;R^{n\times p}$ will be drawn from a multivariate normal distribution with a mean vector of 0 and a variance-covariance matrix $\Sigma \in R^{p\times p}$ with Σ_*ij*_ equal to the correlation coefficient between covariate x_i_ and x_j_ (Table A in S1 File). We will consider ***X*** as random rather than fixed in each simulation experiment. Given ***X***, we will generate *Y* = ***X****β* + *ϵ*, where *ϵ* is assumed to follow an n-variate normal distribution with a zero-mean vector and variance-covariance matrix *σ*^2^*I*_*n*_, *i*.*e*. *ϵ* ~ *N*(0,*σ*^2^*I*_*n*_) with *I*_*n*_ the *n* × *n* identity matrix; β is the true regression coefficient vector with some elements equal to 0 (see Table 1). Henceforth, covariates with nonzero coefficients are called *signal* variables, while those with zero coefficients are called *noise* variables. We will consider six values of theoretical *R*^2^ namely 0.20, 0.30, 0.50, 0.71, 0.80 and 0.90 which correspond to the SNR of 0.25, 0.42, 1.00, 2.5, 4.00 and 9, respectively. For a given value of SNR, vector of true regression coefficients (*β*) and covariance matrix (Σ), the residual variance *σ*^2^ will be calculated as [22]

$$\sigma^{2}=\frac{Var\left(X^{T}\beta \right)}{SNR}=\frac{\beta^{T}\Sigma \beta }{SNR}$$

#### 2.3.2 True regression coefficients considered

It is well documented in the statistical literature that methods behave differently under specific settings of true regression coefficients (*β*). In this study, we will consider four settings as shown in Table 1. The first set, denoted by *β*_*A*_ was investigated by [20] and aims to be more realistic since in real life there exists a mixture of variables with strong, medium and weak effects. The second set *β*_*B*_ is a modification of *β*_*A*_ with one large effect and several smaller effects. Small nonzero regression coefficients were intentionally allowed to investigate the tendency of procedures to reduce false negative results. The third and fourth sets (*β*_*C*_ and *β*_*D*_) are generally not realistic but are often used to study the theoretical properties of methods. Bertsimas and co-authors [21] used *β*_*C*_ and found that the lasso failed to distinguish between zero and nonzero components when the two components were relatively highly correlated. Hastie and co-authors [22] investigated *β*_*D*_ in low-dimensional settings (n = 100, p = 10, *ρ* = 0.35) and found that the relaxed lasso performed best in prediction both in low and high SNR. Therefore, it is important to investigate the properties of other methods such as nonnegative garrote and adaptive lasso under these settings.

The seven nonzero coefficients in *β*_*C*_ are distributed at (roughly) equally-spaced indices between 1 and *p*, and the rest are equal to 0, in order to investigate situations in which zero and nonzero components are weakly and strongly correlated. The first seven coefficients of *β*_*D*_ are nonzero, while the rest are 0. This will enable us to investigate situations where correlations between signal covariates are high. Since the residual variance is a function of SNR, covariance structure and effect size, as described in subsection 2.3.1, it is necessary to adapt the residual variance for each vector of effect size in order to design models with a specified explained variation.

#### 2.3.3 Number of covariates, correlation structure and sample size

*Number of covariates (p)*. The results of variable selection methods are sensitive to the proportion of zero components. For instance, a simulation study conducted by [5, 23] revealed that the subset selection yielded good predictions when the true data generating model contained fewer nonzero coefficients. In order to compare methods on an equal footing, two different sets of covariates will be considered with a fixed number of nonzero components. We will consider a situation where the number of zero and nonzero components is approximately equal (7 zero and 8 nonzero). In an additional analysis, we will investigate the effect of a larger number of zero components (23 zeros and 8 nonzeros). This implies that the latter will not be executed in a full factorial design but considered as part of the additional analysis as described in section 2.

*Correlation structure (C) and multicollinearity*. We will evaluate the correlation structure studied by [20] in order to compare the results. The correlation coefficients for *p* = 15 covariates are displayed in the lower triangular panel of Table A in the S1 File. It is evident that many covariates are uncorrelated; which will enhance the understanding and interpretability of the simulation results [24]. In addition, we will consider the autoregressive (AR) correlation structure where the correlation between covariates x_*i*_ and x_*j*_ is calculated by *ρ*^|*i*−*j*|^ with *ρ* ∈ {0.3, 0.8}. This will allow us to investigate the performance of methods in instances when a signal variable is weakly (*ρ* = 0.3) and strongly (*ρ* = 0.8) correlated with a noise variable. This type of correlation is often used in simulation studies that compare methods, as demonstrated by [5, 21, 22]. Besides, we will use the correlation structure of 13 covariates from the body fat dataset [25], as shown in the upper triangular panel of Table A in the S1 File. Since we are evaluating 15 variables, we will consider the remaining two variables (x14 and x15) uncorrelated with the other variables. A high degree of multicollinearity is expected when the correlation structure of body fat data is used; given that some variables exhibit VIF>10, hence the regression coefficients are likely to be poorly estimated. On the other hand, moderate collinearity is expected when the AR(ρ = 0.8) correlation structure is used, while low collinearity is expected when the AR(ρ = 0.3), and [20] correlation structures are used (Table A in S1 File).

*Sample size (n)*. Numerous challenges are associated with a small sample size relative to the number of parameters to be estimated. These include, (i) an increased risk of excluding important variables when variable selection is conducted, (ii) classical variable selection methods have low power to select important covariates, which can lead to poor predictive performance when evaluated in new data, (iii) internal validation of models is done inefficiently, and (iv) the tuning parameters of penalized regression methods are estimated with large uncertainty [26–29]. When the aim is to build a model and the model-building process involves variable selection, the sample size required should be adequate. This depends on other factors such as the correlation structure of covariates and the strength of effects [30]. A pragmatic approach based on simulation studies such as a range of 10 to 25 observations per regression parameter estimated (OPP) has been recommended [24, 30–32] to ensure that: important variables are included in the model, regression coefficients are accurately estimated, and to avoid serious overfitting. In this study, we will consider the development sample sizes of *n* = (100, 400, 800) with a minimum and maximum sample size of 100 (approx. 7 OPP) and 800 (approx. 53 OPP), respectively, for *p* = 15 covariates. Including a smaller sample size will allow us to elucidate its effects on variable selection and other aspects of model-building. The main analysis will vary n = (100, 400) factorially with other simulation parameters. To shed light on the properties of variable selection strategies on a large sample size, we will conduct additional analysis with *n* = 800 in combination with a subset of the simulation parameters as explained in section 2 on additional analysis.

*Test dataset and number of simulations*. To quantify the performance of fully specified prediction models, test datasets will be independently generated using the same design as that of training datasets, with the assumption that both datasets originate from the same underlying population. Studies show that a small test dataset is unreliable, inaccurate, and biased and should be avoided when making decisions on whether to discard or recommend prediction models [33, 34]. Therefore, the size of the test dataset should be adequate to reduce uncertainties in performance measures when drawing reliable conclusions [29]. As such, we will consider a sample of size *n*_*test*_ = 100,000.

Based on several reviews of simulation studies, a formal justification for the number of simulation repetitions used is hardly provided, even though it plays an important role in the calculation of the Monte Carlo error [16, 35]. The number of simulation repetitions depends on the desired accuracy of an estimate of interest. To get a rough estimate of the number of repetitions, we used the model error (ME) described in section 5 of the S1 File from the full least-squares model (reference model) as a summary statistic of our interest and decided that the Monte Carlo standard error (MCSE) of ME should be lower than 0.005 for better precision. Since the variance of ME was unknown, we performed a small simulation to obtain its realistic estimate as recommended by [35]. We used: p = 15 covariates, n = 400, *R*^*2*^ = 0.50, β_A,_ *n*_*test*_ = 100,000 and N = 1,000 simulation repetitions. The estimate of the variance of ME, $Var\left(M\hat{E}\right)$, was 0.010 and we calculated the number of simulation repetitions using the formula [15]:

$$MCSE(ME)=\sqrt{\frac{Var\left(M\hat{E}\right)}{N}}$$

This means that we need at least N = 400 repetitions to achieve *MCSE*(*ME*) ≤ 0.005. (Fig A in the S1 File) shows that as the number of repetitions increases, the standard error of ME decreases. It was evident that using fewer than 400 repetitions led to high variability, but as N becomes larger, the ME stabilizes. Even though the estimated number of repetitions was 400, it was clear that the ME was not stable. Fig A in S1 File suggests that *N* ≥ 1000 repetitions would provide a reasonable ME with better accuracy (MCSE ranging from 0.003 to 0.001) than *N* = 400. The second summary statistic of interest is the inclusion frequencies of variables. We used the best subset selection with the BIC criterion to select variables using the same design, i.e., p = 15, n = 400, *R*^*2*^ = 0.50, and β_A_. Fig B in S1 File shows the inclusion frequencies for the first four variables (other variables are not shown) versus the number of simulation repetitions, with variables x1 and x3 being signal variables and x2 (uncorrelated to the other variables) and x4 being noise variables. The two signal variables were selected in all simulation runs; hence, their inclusion frequencies are 1, whereas the inclusion frequencies of the two noise variables varied depending on the number of simulation repetitions. It was evident that using fewer than 2000 repetitions resulted in high variability in the inclusion frequency of noise variables (especially x2), but as N became larger, the inclusion frequencies stabilized. Based on these results, we will execute *N* = 2,000 simulation repetitions per scenario and for each repetition, all procedures will be fitted, and evaluation metrics computed.

### 2.4 Estimands and other targets

The estimands of our interest are: (i) the selection status of each covariate (i.e., whether a variable is selected or not) and identification of the true model, (ii) model prediction errors and (iii) shrinkage factors for each regression estimate from penalized and post-estimation shrinkage methods

### 2.5 Methods

#### Ethics committee(s)/institutional review board(s)

This is a protocol for methodological research that will be carried out through a simulation study that does not require any approval from an ethics committee or institutional review board. As such, an approval letter is not necessary.

#### Variable selection and post-estimation shrinkage methods

In penalized likelihood procedures, the nonnegative garrote, lasso, adaptive lasso, and relaxed lasso will be evaluated, while the best subset selection and backward elimination will be considered in traditional variable selection strategies. In post-estimation shrinkage methods, we will consider global [10] and parameterwise [9] shrinkage factors as well as a method proposed by [5]. We will also include and extend the proposed methods for estimating shrinkage factors. As benchmarks, we will compute an oracle estimator (which is the least-squares estimator of the true submodel with seven signal variables) and the full model model with all covariates. Each method is described in section 4 of the S1 File.

### 2.6 Performance measures

Model performance is quantified with respect to the correct inclusion and exclusion of variables and prediction error.

#### 2.6.1 Inclusion and exclusion of variables

*Measure 1a and 1b*: *False positives and false negatives rates*. For each scenario, we will report false positive rates (FPR) and false negative rates (FNR) for individual variables as well as overall false positive and false negative rates for a variable selection approach as described in section 5 of the S1 File. Graphical representations will be used to compare the overall FPR and FNR for all approaches. For example, a plot of FNR against SNR may allow us to uncover the relationship between FNR and SNR and ease the comparison of different approaches.

*Measure 2*: *Classification of selected models*. Using the false positive and false negative rates alone is less informative since it filters a large amount of information into a single number, hence the need for a simple classification that provides more relevant information about models selected in each simulation run. Based on the inclusion and exclusion of variables obtained by using 15 covariates (7 nonzero and 8 zero components), we intend to derive a multicategory response variable named “model category” as shown in Table 2, where: a *true model* is a model that selects the covariates that generated the outcome variable; an *under-selection model* is a model that correctly selects 5 or 6 out of 7 signal variables while excluding all 8 noise variables; an *over-selection model* is a model that identifies all 7 signal variables but includes at most two noise variables; an *almost-real model* is a model that excludes at most two signal variables and includes one or two noise variables, and a *wrong model* is a model that does not belong to the aforementioned model categories. We will calculate the probability of each “model category” in each scenario for different selection approaches and compare the results using a graphical representation. For example, we will plot the probabilities of selecting the true model against SNR (see an example in [22], Fig 6).

**Table 2:** Classification of selected models for 15 covariates (Taken from [24]).

Category	Model Category	# of SV* excluded	# of NV* included
**1**	True	0	0
**2**	Under-selection	1 or 2	0
**3**	Over-selection	0	1 or 2
**4**	Almost-real	1 or 2	1 or 2
**5**	Wrong	Models which cannot be classified in category 1, 2, 3 or 4	

*SV and NV denote signal and noise variables, respectively

#### 2.6.2 Prediction evaluation metrics

Prediction error (PE) refers to the average error in predicting the outcome *Y* from covariate ***X*** for new observations that are not used in building the prediction model [5]. The definition of PE and its estimates differ depending on whether ***X*** is fixed or random and a substantial difference can be observed especially when *n* is small relative to the number of variables [36]. Here, we will estimate the PE of ***X*** random which is more sensible in observational studies because ***X*** is often collected in an uncontrolled setting. We intend to compare the predictive accuracy of all procedures using model error (*ME*) (as conducted by [5]) and investigate the variability of ME within and between scenarios as described in section 5 of the S1 File.

## 3. Final remarks

It is well-known that many simulation studies are often poorly designed, analyzed, and reported [16]. In designing a simulation study, an experienced researcher can easily choose relevant parameters that impact the model results in their favor. This is more prevalent when a preferred method is being evaluated, and it is important to reduce the degree of bias by attempting to design a neutral comparison study which disregards the superiority or inferiority of a particular method [18, 19]. Since we intend to compare several procedures that have been in existence for many years, we have no preferred method. Furthermore, to improve the design of the study, we sought the views of interested STRATOS members and resolved to publish the protocol before conducting the simulation study. We consider this an important step toward a neutral comparison study, whose principal concepts are important and relevant, but whose implementation is often very difficult in practice.

## Supporting information

S1 File Comparison of variable selection procedures and investigation of the role of shrinkage in linear regression-protocol of a simulation study in low-dimensional data. **Table A in S1 File**. Spearman correlation coefficients from body fat (C3) (upper triangular panel) with two additional uncorrelated variables (x14 and x15). In the lower triangular panel are correlation coefficients (C1) used by [3] where blank spaces represent zero correlation coefficients. Variance inflation factors are given for the four correlation structures C1, C2, C3 and C4. **Table B in S1 File**. The Q values for each combination of correlation structures and true regression coefficients. **Fig A in S1 File**. The full least-squares model error (ME) with one standard error band for different number of simulation repetitions ranging from 100 to 5000 by 100. The model errors for different simulation repetitions differ slightly (ranging from 0.250 to 0.269), but the standard errors differ dramatically (ranging from 0.010 to 0.001 for N = 100 and 5000, respectively). **Fig B in S1 File**. The best subset selection with the BIC criterion for settings n = 400, C1, and βA. The inclusion frequency (of 4 out of 15 variables) with one standard error band was calculated for various numbers of simulation repetitions, ranging from 100 to 5000 by 100. Variables x1 and x3 are signal variables, while variables x2 (uncorrelated to the other variables) and x4 are noise variables. The inclusion frequencies of signal variables are 1 while the inclusion frequencies of noise variables vary depending on the number of simulation repetitions. **Fig C in S1 File**. Shrinkage behaviour of the nonnegative garrote (left panel) and the lasso (right panel) for the special setting where the columns of X are orthogonal. The estimate of each procedure (solid line) is plotted against the OLS estimate. The dashed line is the line of equality. Adapted from [6].
(DOCX)
