# Supplementary material for: Comparison of variable selection procedures and investigation of the role of shrinkage in linear regression-protocol of a simulation study in low-dimensional data
Source: PLoS One. 2022 Oct 3;17(10):e0271240. doi: 10.1371/journal.pone.0271240 (PMC9529280; doi:10.1371/journal.pone.0271240)
Supplement: S1 File — Table A in S1 File. Spearman correlation coefficients from body fat (C3) (upper triangular panel) with two additional uncorrelated variables (x14 and x15). In the lower triangular panel are correlation coefficients (C1) used by [3] where blank spaces represent zero correlation coefficients. Variance inflation factors are given for the four correlation structures C1, C2, C3 and C4. Table B in S1 File. The Q values for each combination of correlation structures and true regression coefficients. Fig A in S1 File. The full least-squares model error (ME) with one standard error band for different number of simulation repetitions ranging from 100 to 5000 by 100. The model errors for different simulation repetitions differ slightly (ranging from 0.250 to 0.269), but the standard errors differ dramatically (ranging from 0.010 to 0.001 for N = 100 and 5000, respectively). Fig B in S1 File. The best subset selection with the BIC criterion for settings n = 400, C1, and βA. The inclusion frequency (of 4 out of 15 variables) with one standard error band was calculated for various numbers of simulation repetitions, ranging from 100 to 5000 by 100. Variables x1 and x3 are signal variables, while variables x2 (uncorrelated to the other variables) and x4 are noise variables. The inclusion frequencies of signal variables are 1 while the inclusion frequencies of noise variables vary depending on the number of simulation repetitions. Fig C in S1 File. Shrinkage behaviour of the nonnegative garrote (left panel) and the lasso (right panel) for the special setting where the columns of X are orthogonal. The estimate of each procedure (solid line) is plotted against the OLS estimate. The dashed line is the line of equality. Adapted from [6]. (DOCX) [file pone.0271240.s001.docx]

**Supplementary Material**

**Comparison of variable selection procedures and investigation of the role of shrinkage in linear regression ­­­-protocol of a simulation study in low dimensional data**

Edwin Kipruto^1^, Willi Sauerbrei^1^

^1^Institute of Medical Biometry and Statistics, Faculty of Medicine and Medical Center - University of Freiburg, Germany

## Influence of other related designs

Sample size (n), number of variables (p), effect sizes, correlation structure and noise variance are important parts of the simulation design. It has been shown that the performance of methods highly depends on the signal-to-noise ratio (SNR)—the ratio between the variance of the linear predictor and noise variance [1]. For example, in a simulation study comparing the best subset selection with the lasso and forward stepwise, it was established that the lasso gives better predictive performance than the best subset selection in the low SNR range and worse performance than the best subset selection in the high SNR range [1, 2]. As such, it is advantageous to borrow some ideas from simulation designs that were carefully designed and used in earlier related studies to reduce unintentional bias. For instance, in a simulation study that sought to explore the value of cross-validation, shrinkage and backward elimination (BE) with varying significance levels, the researchers used a design with 15 variables, out of which only seven had an effect on the outcome, and a relatively simple correlation structure where some correlations ranged from 0.3 to 0.7, but most correlations were 0. In addition, they considered an SNR of 1 and 2.5, corresponding to a theoretical *R^2^* of about 0.5 and 0.7, respectively [3]. Their study aimed to assess whether global or parameterwise shrinkage could improve the prediction of selected models using backward elimination and compared the results to the lasso models. The correlation structure used in this design is suitable for understanding and interpretability of the simulation results since some variables were uncorrelated and the effect size used was aimed to be realistic because it was a mixture of strong, medium and weak effects, which are often encountered in practice. However, this design did not consider a wide range of SNR and different settings of correlation structure and effect size. For these reasons, we only included the correlation structure and effect size in our design.

Bertsimas and co-authors [2] designed a simulation study comparing the best subset selection with the lasso and forward stepwise selection in terms of model selection and prediction. They considered (i) SNR in the range of about 2 to 8, which is equivalent to a theoretical *R^2^* of between 0.7 and 0.9, (ii) four different sets of effect size, (iii) correlation between covariates x_i_ and x_j_ was defined by *ρ*^|^*^i-j^*^|^ with *ρ*∈{0.5,0.8,0.9} and (iv) the number of variables considered were 10 and 100 (with only 5 signal variables) for the sample size of 100 and 500 respectively, in their low-dimensional setting. The range of SNR considered was not wide enough to include low SNR since some methods are well known to perform better in low SNR and poorly in high SNR and vice versa [1]. The pairwise correlation of 0.9 is relatively high, which is problematic for parameter estimation and variable selection. In fact, with a pairwise correlation of 0.5 between a signal and a noise variable, the lasso will have trouble selecting variables because the irrepresentability condition does not hold [1]. We included in our design the correlation of *ρ* = 0.8 and two types of effect sizes from this study, called *β_C_* and *β_D_* in Table 1.

It is, however, important to note that the covariance structure often used in simulation studies does not reflect the true complexity of the data encountered in real life. The majority of simulation studies use a covariance structure with a particular structure without noise. This explains why some approaches selected based on simulation study results might perform poorly when applied to real data. For this reason, we incorporated a covariance structure from a real dataset into our design, a concept of plasmode simulation as explained in [4]. A summary of the key elements of the simulation study is presented in Table 1.

## Degree of multicollinearity

Another important aspect that is closely related to correlation in statistical modeling is multicollinearity. This refers to the situation in which two or more covariate variables are exactly or approximately linearly dependent. In stepwise variable selection, multicollinearity can cause predictors to compete, making the selection of signal variables arbitrary [5]. In a severe case of multicollinearity, it is difficult to determine the importance of a given explanatory variable because the effects of explanatory variables are confounded. Using each of the correlation structures, we calculated the variance inflation factors (VIF), which in ordinary least-squares are the main diagonal elements of the inverse of the correlation matrix of the variables [5] and are displayed in the Table A.

**Table A.** Spearman correlation coefficients from body fat (C3) (upper triangular panel) with two additional uncorrelated variables (x14 and x15). In the lower triangular panel are correlation coefficients (C1) used by [3] where blank spaces represent zero correlation coefficients. Variance inflation factors are given for the four correlation structures C1, C2, C3 and C4.

|  | x1 | x2 | x3 | x4 | x5 | x6 | x7 | x8 | x9 | x10 | x11 | x12 | x13 | x14 | x15 |
| --- | --- | --- | --- | --- | --- | --- | --- | --- | --- | --- | --- | --- | --- | --- | --- |
| x1 | - | -0.01 | -0.23 | 0.12 | 0.17 | 0.22 | -0.07 | -0.20 | 0.01 | -0.13 | -0.04 | -0.07 | 0.22 |  |  |
| x2 |  | - | 0.52 | 0.80 | 0.90 | 0.87 | 0.93 | 0.84 | 0.83 | 0.70 | 0.78 | 0.75 | 0.70 |  |  |
| x3 |  |  | - | 0.32 | 0.26 | 0.23 | 0.43 | 0.34 | 0.51 | 0.46 | 0.31 | 0.34 | 0.39 |  |  |
| x4 |  |  |  | - | 0.78 | 0.74 | 0.71 | 0.65 | 0.64 | 0.52 | 0.69 | 0.71 | 0.72 |  |  |
| x5 |  |  |  |  | - | 0.90 | 0.81 | 0.72 | 0.71 | 0.56 | 0.74 | 0.68 | 0.65 |  |  |
| x6 | 0.5 |  |  |  |  | - | 0.85 | 0.73 | 0.72 | 0.51 | 0.67 | 0.59 | 0.59 |  |  |
| x7 |  |  |  |  | 0.3 |  | - | 0.88 | 0.80 | 0.63 | 0.74 | 0.68 | 0.59 |  |  |
| x8 |  |  |  |  |  |  |  | - | 0.77 | 0.61 | 0.74 | 0.67 | 0.50 |  |  |
| x9 |  |  |  | 0.5 |  |  |  |  | - | 0.73 | 0.63 | 0.63 | 0.65 |  |  |
| x10 |  |  |  |  |  |  |  | 0.7 |  | - | 0.53 | 0.56 | 0.65 |  |  |
| x11 |  |  |  |  |  | 0.7 |  |  |  |  | - | 0.76 | 0.60 |  |  |
| x12 |  |  | 0.5 |  |  |  |  |  |  |  |  | - | 0.64 |  |  |
| x13 |  |  |  |  | -0.7 |  |  |  |  |  |  |  | - |  |  |
| x14 |  |  |  |  |  |  | 0.5 |  |  |  |  |  |  | - |  |
| x15 |  |  |  |  |  |  |  |  |  |  |  |  |  |  | - |
| VIF(C1) | 2.0 | 1.0 | 1.3 | 1.3 | 2.6 | 3.8 | 1.7 | 2.0 | 1.3 | 2.0 | 2.9 | 1.3 | 2.3 | 1.4 | 1.0 |
| VIF(C2) | 1.1 | 1.2 | 1.2 | 1.2 | 1.2 | 1.2 | 1.2 | 1.2 | 1.2 | 1.2 | 1.2 | 1.2 | 1.2 | 1.2 | 1.1 |
| VIF(C3) | 2.8 | 4.6 | 4.6 | 4.6 | 4.6 | 4.6 | 4.6 | 4.6 | 4.6 | 4.6 | 4.6 | 4.6 | 4.6 | 4.6 | 2.8 |
| VIF(C4) | 1.9 | 34.7 | 2.9 | 3.8 | 11.2 | 9.4 | 12.3 | 6.6 | 4.7 | 3.0 | 3.5 | 3.3 | 3.4 | 1.0 | 1.0 |

## Number of simulation repetition


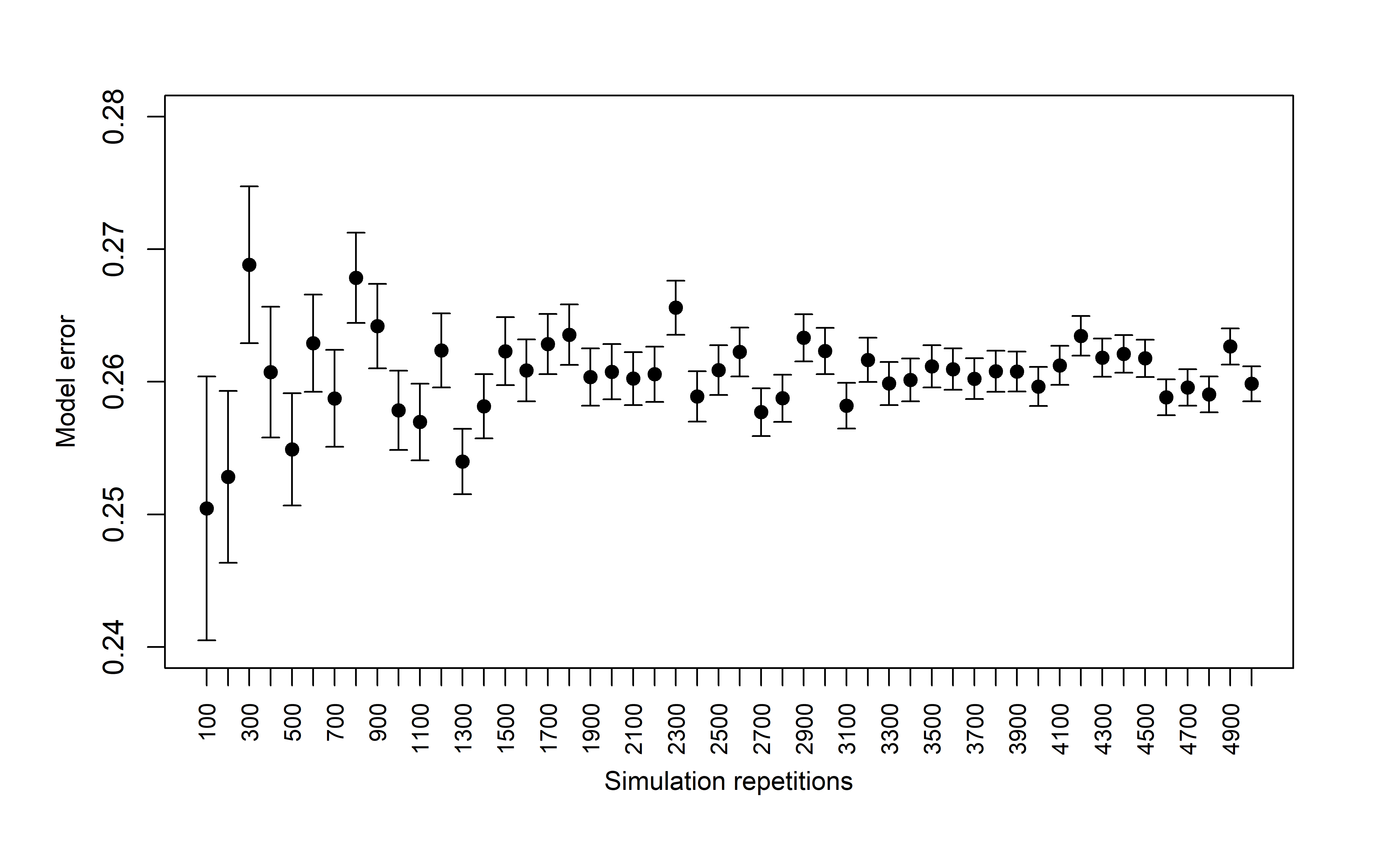


**Figure A.** The full least-squares model error (ME) with one standard error band for different number of simulation repetitions ranging from 100 to 5000 by 100. The model errors for different simulation repetitions differ slightly (ranging from 0.250 to 0.269), but the standard errors differ dramatically (ranging from 0.010 to 0.001 for N = 100 and 5000, respectively).


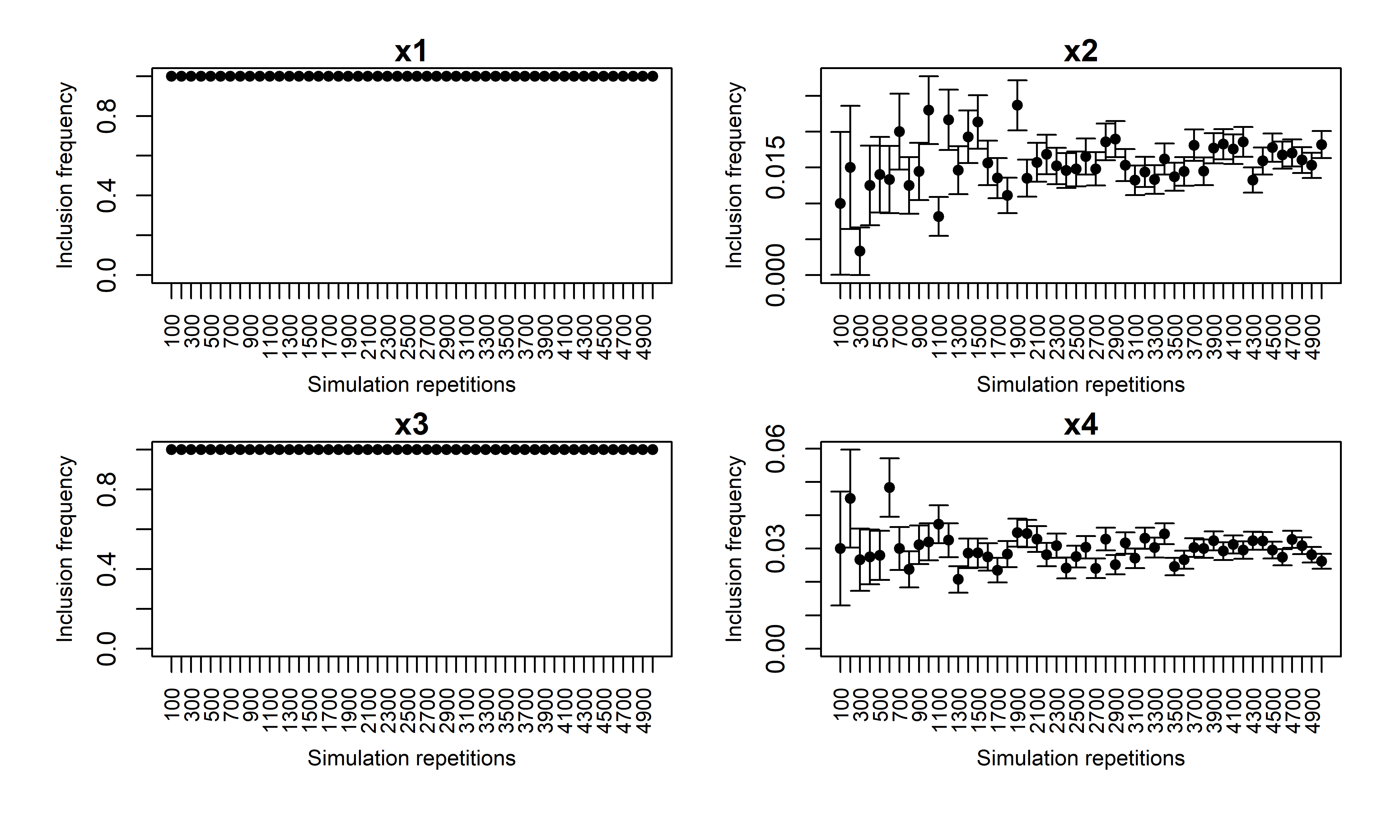


**Figure B.** The best subset selection with the BIC criterion for settings n = 400, C1, and βA. The inclusion frequency (of 4 out of 15 variables) with one standard error band was calculated for various numbers of simulation repetitions, ranging from 100 to 5000 by 100. Variables x1 and x3 are signal variables, while variables x2 (uncorrelated to the other variables) and x4 are noise variables. The inclusion frequencies of signal variables are 1 while the inclusion frequencies of noise variables vary depending on the number of simulation repetitions.

# Additional details on methods

### 4.1 Linear models

To clearly understand the concepts of variable selection methods, we illustrate with a full linear model with continuous covariates. Let $Y=\left( y_{1},\ldots,y_{n} \right)^{T}$ be a vector of continuous outcome variable and$x_{j}=\left( x_{1j},\ldots,x_{nj} \right)^{T}$, $j=1,\ldots,p$ be linearly independent covariates. Let $\boldsymbol{X}=[x_{1},\ldots,x_{p}]$ be the covariate matrix and assume that $Y$ is mean-centered and $x_{j}$ is mean-centered and scaled by its standard deviation in order to have a zero mean and unit variance so that the intercept is zero and $X^{T}X/n$is the sample correlation matrix of the original covariate variables. Then the traditional linear regression model is defined as $y_{i} =\beta_{1}x_{i1}+\ldots+ \beta_{p}x_{ip}+ \epsilon_{i}$ which can be written in matrix form as $Y=\boldsymbol{X}\beta+\epsilon,$ where $\epsilon_{i}$is the random noise assumed to be iid $N(0,\sigma^{2})$. The least-squares estimators$\hat{\beta}^{OLS}=\left( \hat{\beta}_{1}, ..,\hat{\beta}_{P} \right)^{T}$ are unbiased estimators for $\beta$. In practice, $\hat{\beta}^{OLS}$ are estimated by minimizing the residual sum of squares (RSS):

$$\hat{\beta}^{OLS}=\text{arg }\text{min}_{\beta}\frac{1}{2n} {\sum_{i=1}^{n} \left( y_{i}-\sum_{j=1}^{p} \beta_{j}x_{ij} \right)}^{2}$$

The challenge with this approach is that it has too many parameters to estimate when $p$ is large, thus producing a higher prediction error on the new data and is often difficult to interpret [5]. Furthermore, variables without effect need to be measured for practical use and may be costly. However, overfitting can be mitigated by eliminating noise variables. On the contrary, when variables with effects are not included, the model may not fit the data well. To get a compromise between these situations, a good variable selection strategy that produces a simpler and more accurate model compared to the model with all variables is required [6, 7]. Moreover, when the number of covariates is almost equal to the number of observations ($p\approx n$) or in a high degree of multicollinearity the least-squares estimate is known to be extremely variable. Furthermore, when$p>n$, least-squares estimates have no unique solution (the variance is infinite), hence the method cannot be used at all [8, 9]. Penalized regression techniques that shrink regression estimates towards zero while forcing some of the estimates to be exactly equal to zero, hence performing variable selection, were proposed to improve least-squares estimators and are discussed in subsequent sections.

### 4.2 Nonnegative garrote

The original nonnegative garrote (NNG) estimator [10] consists of three parts: the initial estimation of least squares estimates $\hat{\beta}^{OLS}$ from the full model (model with all variables), the selection of the tuning parameter λ and the estimation of nonnegative shrinkage factors$c = \left( c_{1}, . . . , c_{p} \right)^{T}$. The process of obtaining shrinkage factors is as follows. First, obtain the OLS estimates $\hat{\beta}^{OLS}$ from the full linear regression model. Secondly, calculate $x_{ij}^{*}=\hat{\beta}_{j}^{OLS}x_{ij}$ and minimize the NNG objective function. Minimizing the NNG objective function is equivalent to minimizing the lasso objective function (see section 4.3 on the lasso) with non-negativity constraints on the regression parameters [10, 11].

$$\hat{c} (\lambda)=\text{arg }\text{min}_{c}\frac{1}{2n} {\sum_{i=1}^{n} \left( y_{i}-\sum_{j=1}^{p} c_{j}{x^{*}}_{ij} \right)}^{2}+\lambda\sum_{j=1}^{p} c_{j}, c_{j}\geq0, \lambda\geq0$$

After obtaining$\hat{c}_{j}(\lambda)$, the NNG estimate is calculated as $\hat{\beta}_{j}^{NNG}(\lambda)=\hat{c}_{j}\hat{\beta}_{j}^{OLS}$, thus, the original NNG estimate of the regression coefficient is a scaled version of the least square estimate*.* In the special case where the columns of ***X*** are orthogonal *i.e.* $\boldsymbol{X}^{T}\boldsymbol{X}=I_{n},$ the shrinkage factors can be obtained using $\hat{c}_{j}(\lambda)= \left( 1-\frac{\lambda}{\left( \hat{\beta}_{j}^{OLS} \right)^{2}} \right)_{+}$ where ${(z)}_{+}=\max\left( z, 0 \right).$ Figure C (left panel) shows the form of this function. This indicates that the shrinkage factors for regression coefficients whose least square estimate is large in absolute terms in the full model will have shrinkage factors close to 1, hence the NNG estimate will be approximately equal to the OLS estimate. For a noise covariate, the least square estimate is likely to be close to zero and as a result, the shrinkage factor can be exactly zero as shown on the left panel of Figure C. In a general (non-orthogonal) setting of design matrix **X**, the main ideas still hold approximately [6].


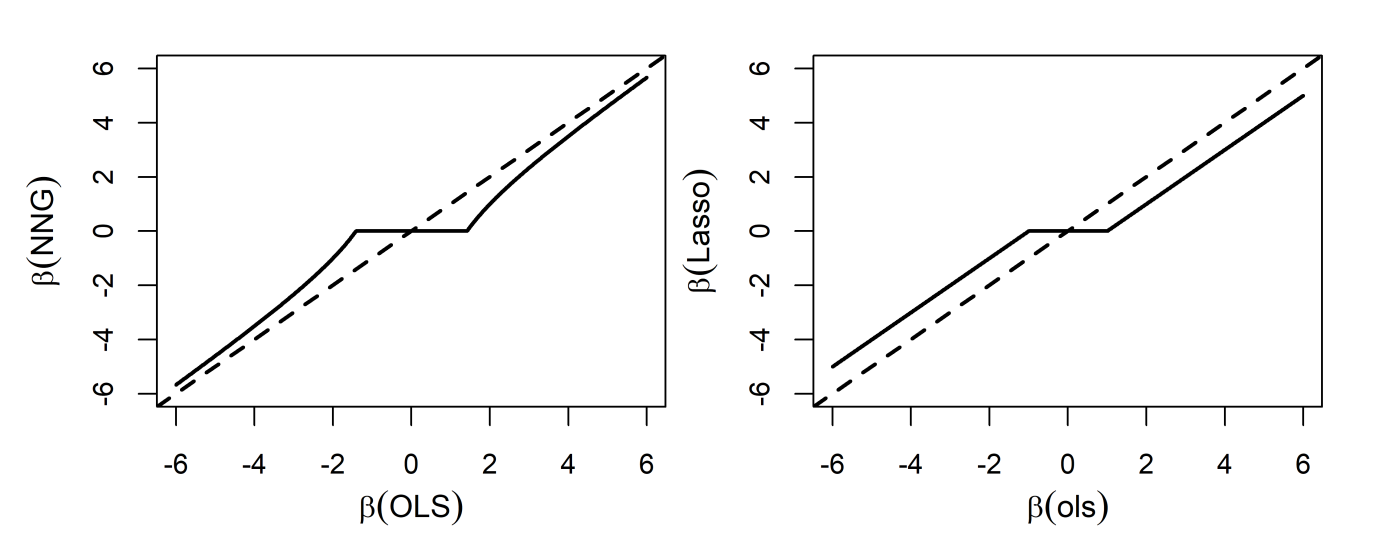


**Figure C.** Shrinkage behaviour of the nonnegative garrote (left panel) and the lasso (right panel) for the special setting where the columns of **X** are orthogonal. The estimate of each procedure (solid line) is plotted against the OLS estimate. The dashed line is the line of equality. Adapted from [6].

The tuning parameter $\lambda$ controls the amount of shrinkage that is applied to the regression coefficients. When $\lambda=0,$ the penalty term has no effect and all $\hat{c}_{j}(\lambda=0)=1,$ hence NNG estimates are equal to OLS estimates ($\hat{\beta}^{NNG}(\lambda=0)= \hat{\beta}^{OLS}$). Conversely, when $\lambda\to\infty,$ the impact of the penalty term is high and all $\hat{c}_{j}=0$. Therefore, depending on the value of$\lambda$, NNG can produce a model with any number of variables, which implies that its performance critically depends on the tuning parameter. It has also been shown that NNG is variable selection consistent in that as the sample size $n\to\infty,$ the probability that the procedure correctly identifies the set of signal variables ($\beta_{j}\neq0$) approaches one, provided that $\lambda$ is appropriately chosen and the initial estimates are consistent. For instance, initial estimates obtained from least-squares when $p<n$and those based on other estimates such as ridge, lasso or elastic net [11, 12]. Lastly, unlike lasso or ridge, NNG is scale invariant in that if the scale used to measure individual covariates is changed, then the NNG estimates change inversely proportional to the changes in variable scales [10].

### 4.3 Lasso

Since there is a vast literature on the lasso, we briefly present some key concepts and issues. For the linear regression problem, the lasso estimator is the value that minimizes the objective function

$$\hat{\beta}^{lasso}(\lambda)=\text{arg }\text{min}_{\beta}\frac{1}{2n} {\sum_{i=1}^{n} \left( y_{i}-\sum_{j=1}^{p} \beta_{j}x_{ij} \right)}^{2}+\lambda\sum_{j=1}^{p} {|\beta}_{j}|, \lambda\geq0$$

where $\left| \beta\right|_{1}=\sum_{j=1}^{p} |\beta_{j}|$is the $\mathcal{l}_{1}$penalty on $\beta$ that shrinks the regression coefficients toward zero, while setting some of the coefficients to be exactly equal to zero, thus producing sparse interpretable models [6]. The lasso is probably highly popular due to the availability of fast algorithms for the estimation of its solution, especially in high dimensional data [13]. Although lasso has shown success in many situations, it has some drawbacks. First, it suffers from variable selection inconsistency unless the design matrix ($\boldsymbol{X}$) satisfies strong assumptions, the so-called irrepresentable condition as described in subsection 4.3.1. Even if the lasso is variable selection consistent, it is not efficient in estimating coefficients of nonzero components since the resulting value of λ that yielded consistency over-shrinks the nonzero coefficients, which can be detrimental to prediction due to an excessive amount of bias. Secondly, by design, lasso shrinks small and large nonzero coefficients equally, as shown on the right panel of Figure C, and this also holds approximately in general settings, implying that asymptotically it cannot produce unbiased estimates even for large coefficients. Third, in high-dimensional data$(p>n)$, the lasso selects at most $n=min(n,p)$ variables, this property is called saturation of the lasso solution [16], which is an important caveat if the true data generating model consists of more than $n$ covariates [12, 17, 18]. Fourth, the lasso with a prediction-optimality penalty has been shown to select many noise variables, which is an undesirable property when the aim is to understand the association between the outcome and covariate variables [3, 19, 20]. Several modifications of the lasso penalty have been proposed to reduce the number of noise variables and estimation bias, and they are discussed in subsections 4.4 and 4.5.

### 4.3.1 Irrepresentable condition of the lasso

Zhao and Yu [14] investigated the so-called irrepresentable condition, which is necessary for the lasso to consistently select the true model. The irrepresentable condition depends on the correlation between signal and noise variables as well as the correlation within signal variables. If these correlations are strong, the irrepresentable condition does not hold [12, 14, 15]. Without loss of generality, assume that the first *k* elements of the regression coefficients are nonzero, i.e., $\beta_{j}\neq0$ for *j = 1,…,k* and the rest are zero, i.e., $\beta_{j}=0$ for *j = k+1,…,p.* Let $\Sigma= \left( \begin{matrix} \Sigma_{1,1} & \Sigma_{12} \\ \Sigma_{21} & \Sigma_{22} \end{matrix} \right)$ be the variance-covariance matrix of the design matrix **X,** where $\Sigma_{11}$ and $\Sigma_{22}$ are the variance-covariance matrices of signal and noise variables, respectively, and $\Sigma_{12}=\Sigma_{21}^{T}$ is the covariance between signal and noise variables. The irrepresentable condition is fulfilled if

$${Q= ||\Sigma_{21}\Sigma_{11}^{-1}S||}_{\infty}<1$$

where $S=\left( sign\left( \beta_{1} \right),\ldots,sign\left( \beta_{k} \right) \right)^{T}$ is a sign vector of regression coefficients of signal variables such that $sign\left( \beta_{j} \right)=1$ if $\beta_{j}>0$ or $sign\left( \beta_{j} \right)=-1$ if $\beta_{j}<0$ and ${||x||}_{\infty}=\max_{j} |x^{(j)}|$ where $x$ is a vector. More details on irrepresentable conditions are found in [14, 15].

We calculated the quantity $Q$ using each of the correlation structures and signs of the true regression coefficients, as shown in Table B. This gave us a rough idea of which correlation structures and regression coefficients the lasso would be inconsistent in model selection. The irrepresentable condition will hold when correlation structure C2 and any of the four regression coefficients are used since all the $Q$ values are significantly smaller than 1 (Table B). However, due to a high degree of multicollinearity, the irrepresentable condition will fail for correlation structure C4 with any of the regression coefficients. In addition, the irrepresentable condition may fail when correlation structure C3 and regression coefficients $\beta_{A}, \beta_{B}$ or $\beta_{C}$ are used since their corresponding $Q$ values are close to 1. This is because the signal and noise variables were intentionally allowed to be highly correlated. We expect the lasso to perform well in selecting the true model when $\beta_{D}$ and any of the correlation structures C1, C2 and C3 are used since the Q values are far away from 1.

**Table B.** The Q values for each combination of correlation structures and true regression coefficients

| Correlation  structure | True regression coefficients | | | |
| --- | --- | --- | --- | --- |
|  | $\beta_{A}$ | $\beta_{B}$ | $\beta_{C}$ | $\beta_{D}$ |
| C1 | **1.20** | **1.20** | **1.20** | 0.54 |
| C2 | 0.55 | 0.55 | 0.55 | 0.30 |
| C3 | 0.98 | 0.98 | 0.98 | 0.80 |
| C4 | **1.15** | **1.15** | **1.15** | **1.01** |

### 4.4 Adaptive lasso

Adaptive lasso (Alasso) is an extension of the lasso [12]. It modifies the lasso penalty by assigning different weights to different coefficients, where the role of weights is to impose severe shrinkage on small coefficients while large coefficients are hardly shrunken. Therefore, its shrinkage behavior is similar to the nonnegative garrote presented in Figure C (see Figure 1 in [12] for clarity), which is a desirable property, especially when the regression estimates are of primary interest. As a result, the estimation bias of nonzero coefficients experienced in lasso is reduced and the accuracy of identifying important covariates is improved [12]. The Alasso coefficients are obtained by minimizing the objective function

$$\hat{\beta}^{Alasso}(\lambda,\gamma)=\text{arg }\text{min}_{c}\frac{1}{2n} {\sum_{i=1}^{n} \left( y_{i}-\sum_{j=1}^{p} \beta_{j}x_{ij} \right)}^{2}+\lambda\sum_{j=1}^{p} {w_{j}|\beta}_{j}|, \lambda\geq0$$

where the data-dependent weights $w_{j}=1/\left| \hat{\beta}_{j}^{init} \right|^{\gamma}$are functions of the initial estimator $\hat{\beta}_{j}^{init}$, and $\gamma>0$is a positive constant. When $\gamma=0,$ all $w_{j}=1$ and adaptive lasso reduces to the lasso

For a fixed number of covariates (*p*), it has been shown that Alasso in theory has an oracle property in terms of selecting the correct model, provided the tuning parameters $(\lambda,\gamma)$ and data-dependent weights are appropriately chosen [12]. This assures two important asymptotic properties. First, as$n\to\infty$, the selected set of covariate variables approaches the true set with probability tending to 1. Secondly, the estimators are asymptotically normal, with the same mean and covariance that they would have by maximum likelihood estimation when the correct submodel is known in advance [12]. It has been postulated that a good variable selection procedure should have continuous shrinkage and oracle properties for the stability of model selection and unbiasedness of estimates for large coefficients [12, 21].

### 4.5 Relaxed lasso

Relaxed lasso (Rlasso) is a variant of the lasso proposed by [19] with the aim of (i) reducing the number of noise variables selected by the lasso when cross-validation is used to select the optimal tuning parameter, (ii) reducing the estimation bias of nonzero coefficients caused by overshrinkage, and (iii) reducing the computational burden experienced by the lasso in sparse, high-dimensional data where the number of covariates grows faster with the number of observations. The relaxed lasso is a two-stage procedure. In the first stage, the lasso is used to select the covariates, while in the second stage, the lasso is again applied but using only the selected covariates from the first stage. The latter is important because it further reduces noise variables that were not eliminated in the first stage and since there is less competition between noise and signal variables, the tuning parameter estimated by cross-validation will be smaller, hence producing less shrunken nonzero coefficients [19, 22].

Let $A_{\lambda}$ be the set of covariates selected by the lasso, then the relaxed lasso estimator is obtained by minimizing [19].

$$\hat{\beta}^{relax}(\lambda,\phi)=\text{arg }\text{min}_{\beta}\frac{1}{2n} {\sum_{i=1}^{n} \left( y_{i}-\sum_{j=1}^{p} {(\beta}_{j}.1_{A_{\lambda}})x_{ij} \right)}^{2}+\lambda\phi\sum_{j=1}^{p} {|\beta}_{j}|, \lambda\geq0, 0\leq\phi\leq1$$

where $1_{A_{\lambda}}$ is an indicator function that takes value 1 if $X_{j}\in A_{\lambda}$ and 0 otherwise, such that

$$\beta_{j}.1_{A_{\lambda}}=\left\{ \begin{aligned} \beta_{j} if X_{j}\in A_{\lambda} \\ 0 if X_{j}\notin A_{\lambda} \end{aligned} \right.$$

A simplified version of the relaxed lasso estimator above is given by $\hat{\beta}^{relax}\left( \lambda, \phi\right)=\phi\hat{\beta}^{lasso}\left( \lambda\right)+\left( 1-\phi\right)\hat{\beta}^{OLS}$ where $\hat{\beta}^{OLS}$ denotes the OLS estimates obtained by regressing Y on $X_{A_{\lambda}}$, padded with zeros to match the zeros of the lasso solution [1]. The role of $\phi$ is to control the amount of shrinkage of coefficients such that when $\phi=1,$ the solution of the relaxed lasso and the lasso are identical. However, when $\phi<1,$ the amount of shrinkage of coefficients in the selected model is reduced compared to the lasso. Similarly, when $\phi=0,$ the relaxed lasso estimators are equivalent to the OLS estimators for the variables selected by the lasso ( *i.e.,* variables in set $A_{\lambda}$). In this case, the solution is only feasible when the sample size is relatively larger than the selected covariates in $A_{\lambda}.$ Otherwise, it would produce a degenerate solution [19].

It has been shown that this approach has the property of variable selection consistency when tuning parameters are obtained via cross-validation [19]. In addition, it is more effective in eliminating noise variables in high-dimensional settings than the lasso, which in turn produces more accurate predictions than the lasso, especially in high SNR, where shrinkage of the strong nonzero components is not necessary [1, 19]. Recently, it was established that generally, relaxed lasso performed very well in prediction compared to lasso, best subset selection, and forward selection across different settings of simulation parameters in low and high-dimensional settings [1].

### 4.6 Tuning parameters for nonnegative garrote, lasso, adaptive lasso, and relaxed lasso

A crucial part of a model-building strategy, especially in penalized likelihood methods, is the selection of the tuning parameters. Several methods for selecting tuning parameters have been proposed, depending on the aim of the analysis. For instance, cross-validation (CV) or Akaike Information Criterion (AIC) are often preferred in prediction modeling because they tend to select models with good prediction performance. On the other hand, tuning parameters from the Bayesian Information Criterion (BIC) are preferred when the aim is to recover the underlying set of signal variables [15]. Therefore, we will compare the performance of the tuning parameters from CV, AIC, and BIC.

**CV, AIC and BIC tuning parameters**

It was reported by [10] that the 10-fold CV was more reliable than leave-one-out CV (LOOCV) in estimating the tuning parameter for the NNG. For this reason, we will estimate $\lambda$ using 10-fold CV. Given the initial estimates ($\hat{\beta}^{init}$), we will evaluate 100 values of $\lambda$ ranging from $\lambda_{max}=\frac{\max\left( \left( \hat{\beta}^{init}\boldsymbol{X} \right)^{T}Y \right)}{n}$ where all shrinkage factors are equal (or almost) to zero to a small fraction of $\lambda_{max}$ given by $\lambda_{min}=\epsilon\lambda_{max}>0$ on a log scale where $\epsilon=0.0001$.

The lasso will be tuned over 100 values of λ ranging from $\lambda_{max}=\frac{\max\left( X^{T}Y \right)}{\boldsymbol{n}}$ to a small fraction of $\lambda_{max}=\epsilon\lambda_{max}$ on a log scale, as per the default in *cv.glmnet* function in glmnet package in R software (see [23] for more details).

The adaptive lasso has two tuning parameters $\lambda$ and $\gamma$ when the initial estimates ${(\hat{\beta}}^{init})$ are available. To find the optimal pair ($\lambda,\gamma)$, two-dimensional 10-fold CV will be used. We will evaluate four values of $\gamma=(0.5, 1, 1.5, 2)$. To obtain the grid of $\lambda$ for adaptive lasso we will proceed as follows. For each value of $\gamma$, we will calculate $\lambda_{max}(\gamma)=\frac{\max\left( \left( \left| \hat{\beta}^{init} \right|^{\gamma}\boldsymbol{X} \right)^{T}Y \right)}{n}$ where all regression estimates are equal (or almost) to zero. In particular, we have $\lambda_{max}= \left( \lambda_{max}\left( 0.5 \right), ..,\lambda_{max}\left( 2 \right) \right)$ and their corresponding $\lambda_{min}= \left( {\epsilon\lambda}_{max}\left( 0.5 \right), ..,\epsilon\lambda_{max}\left( 2 \right) \right)$. Finally, the adaptive lasso will be tuned over 100 values of $\boldsymbol{\lambda}$ ranging from $\lambda_{max}^{*}=max(\lambda_{max}\left( 0.5 \right),\ldots,\lambda_{max}(2))$ to $\lambda_{min}^{*}=min \left( {\epsilon\lambda}_{max}\left( 0.5 \right),\ldots,{\epsilon\lambda}_{max}(2) \right)$ on a log scale. In total, 400 pairs of tuning parameters will be evaluated.

The relaxed lasso has two tuning parameters $\lambda$ and $\phi$. The optimal pair ($\lambda,\phi)$ will be obtained via two-dimensional 10-fold CV. We will evaluate 100 values of $\lambda$ (same grid of $\lambda$ as in lasso) and five values of $\phi=(0, 0.25, 0.5, 0.75 1)$ as per the default in *cv.glmnet* function. In all procedures, tuning will be performed by minimizing mean squared error.

Cross-validation is a classical method for selecting tuning parameters for penalized methods. Alternatively, a simple and computationally efficient approach is to use information criteria, such as the AIC or BIC, which penalize the likelihood by the degrees of freedom of the fitted model [15]. Initially, these information criteria were proposed for models estimated by the maximum likelihood method, but they have been extended to models estimated by the penalized maximum likelihood method [24]. It has been shown that AIC performs like cross-validation with the potential for selecting complex models even in large sample sizes [25, 26, 27], while BIC has a higher tendency to correctly identify the true model than CV or AIC in penalized methods such as SCAD [27]. For a Gaussian linear model, the generalized information criterion (GIC) for the estimated model with fitted values $\hat{y}_{i}$ ($i=1,\ldots,n$) is given by [24]

$$GIC=\frac{RSS}{n\hat{\sigma}^{2}} +\frac{w_{n}}{n}df\left( \hat{Y} \right)$$

where $w_{n}=2$ is AIC and $w_{n}=log(n)$ is BIC. df is the degrees of freedom of the fitted model. When maximum likelihood estimation is used in parametric models, the degrees of freedom is equal to the number of estimated parameters. However, in penalized likelihood methods such as the lasso in low-dimensional settings, df is the number of nonzero estimated parameters [24]. $\hat{\sigma}^{2}$ is the estimate of the residual variance estimated using the full model containing all covariates, while RSS is the residual sum of squares of a selected model [9]. Several modifications of BIC have been proposed for high-dimensional settings. We refer to [28] for tuning parameter selection in high-dimensional data, and [29] for extended BIC.

To estimate the tuning parameters using AIC and BIC for the lasso we proceed as follows. For a grid of 100 values of λ equally spaced on the log scale over [$\lambda_{max}, \lambda_{min}$], we will calculate the lasso penalized regression estimates. This results in a sequence of 100 candidate models. Then, for each model, we will calculate BIC and AIC and select the best model (*i.e.,* the model with the smallest BIC or AIC) from the sequence and evaluate its predictive performance using test data. We will repeat the same procedure for NNG because it also has one tuning parameter like the lasso. The adaptive lasso has two tuning parameters. For each pair of tuning parameters ($\lambda,\gamma$) we will fit the adaptive lasso model, which will result in 400 candidate models, and then calculate the AIC and BIC and select the model with the smallest AIC or BIC. The same procedure for adaptive lasso will be repeated for relaxed lasso because it also has two tuning parameters ($\lambda, \phi$).

### 4.7 Initial estimates for nonnegative garrote and adaptive lasso

The original nonnegative garrote explicitly relies on OLS estimates. In high degree of multicollinearity, the OLS estimates are poorly estimated. Similarly, when the number of covariates is almost equal to the sample size, the OLS estimates are extremely variable [9]. These two phenomena affect NNG. Furthermore, if $p>n$ the OLS estimates do not exist and NNG selection is not possible; a probable reason that NNG is not used in the analysis of high-dimensional data. However, it has been shown that NNG is a flexible approach that can be used with other estimators besides OLS, such as ridge, lasso, and elastic net, so the aforementioned challenges can be avoided [11].

On the other hand, the use of OLS estimates from the full model has been recommended as initial estimates for adaptive lasso except when multicollinearity is of concern, in which ridge regression estimates using optimal tuning parameters were proposed [12]. In addition, the use of lasso regression estimates tuned in a prediction optimality way has been proposed as initial estimates for the adaptive lasso because the lasso often selects too many noise variables [15]. Thus, the adaptive lasso in the second stage is used to reduce the number of false positives selected by the lasso.

For these reasons, we will use OLS, ridge, and lasso regression estimates as initial estimates for nonnegative garrote and adaptive lasso, and compare their performances. The optimal tuning parameter of ridge and lasso will be estimated using 10-fold cross-validation where 100 values of $\lambda$ will be evaluated as per the default in the *cv.glmnet* function in R software.

### 4.8 Best subset selection

This is one of the classical methods of variable selection with a vast amount of literature in statistics and efficient computation algorithms. It involves identifying subsets of $p$ covariates via an exhaustive search that fits the data well, then applying a stopping rule to choose the best-fitting subset of variables, and lastly, estimating regression coefficients of selected variables using least squares as if the selected model was given a priori. The latter might produce biased regression coefficients since the same data was used in the selection of variables and estimation of regression coefficients, hence the need for shrinkage [30]. The estimates of the best-fitting subset of size *k* are obtained by optimizing the objective function:

$$\hat{\beta}^{subset}= \text{ }\text{arg }\text{min}_{\beta}\frac{1}{2n} {\sum_{i=1}^{n} \left( y_{i}-\sum_{j=1}^{p} \beta_{j}x_{ij} \right)}^{2}, subject to \sum_{j=1}^{p} 1(\beta_{j}\neq0)\leq k$$

Where $1(\cdot)$ is an indicator function that takes the value $1$ when a variable is selected and 0 otherwise. In situations where there are too many covariates, the number of subsets grows exponentially and an exhaustive search becomes infeasible. For this reason, it was widely dismissed by the greater statistical community before [31] introduced a pure branch and bound algorithm that is very efficient in selecting the best subset without an exhaustive search. This approach has been implemented in statistical softwares such as the leaps package in R software. Despite its impressive speed, it becomes computationally intractable for a large number of variables ($p>30$). However, it has been shown that the best subset selection problem can be formulated as a mixed-integer optimization (MIO) problem, which can then be solved using hybrids of branch and bound and cutting plane algorithms, thus handling much larger problem sizes [2]. This new development will ease the comparison of the classical best subset with modern variable selection methods in large problem sizes. Even though best subset selection can produce a relatively sparse model (depending on the stopping criteria), it has some weaknesses: (i) it is unstable due to its inherent discreteness, *i.e*., covariates are either retained or dropped from the model [7] and (ii) it can lead to the selection of spurious covariates caused by searching over larger sets of models [9, 32]. The stopping criteria play an important role in determining the size of the final model and are discussed in subsection 4.10.

### 4.9 Backward elimination

Backward elimination (BE) is a computationally efficient alternative to best subset selection since it investigates a much smaller set of models. It begins with the full least squares model containing all covariates and then iteratively removes the least significant covariate one by one, based on a pre-specified stopping criterion [9, 32]. As such, the number of variables selected depends on the stopping criteria. Some algorithms allow the re-inclusion of an omitted variable as well as the removal of an included variable, which no longer provides an improvement in the model fit. Among the classical variable selection methods, BE is generally preferred because it starts with a more plausible overfitted model and drops covariates when it can afford to drop them without worsening the fit of the model [33]. However, like all other stepwise selection methods, BE does not guarantee the best possible model out of all the $2^{p}$ possible models and can only be used in low-dimensional settings because of the necessity of a full model in the first step [9].

### 4.10 Stopping criteria for best subset selection and backward elimination

An algorithm that performs an exhaustive search and returns the best model for each size will be used to implement the best subset selection, where the best model is defined as a model having the smallest RSS for each model size [34]. Several approaches for selecting a single best model have been proposed, among them CV, AIC, and BIC. The AIC, BIC, and various significance levels (0.157, 0.05, 0.01) are often used as stopping criteria for BE. For both methods, we will employ the three commonly used criteria, *i.e.,* CV, AIC, and BIC; the former was chosen to compare the performance of classical and modern methods of variable selection on equal footing. It is important to note that the choice of the criteria depends on the aim of the study. Since our primary interest is descriptive modeling, we prefer simpler models that are more stable and interpretable and can be achieved by BIC [35, 36]. On the other hand, our secondary interest is in predictive models, implying that a complex model which includes weak effect variables may be tolerated, hence CV and AIC are more suitable [26, 32].

### 4.11 Post-estimation shrinkage methods

**4.11.1 Global shrinkage**

van Houwelingen and le Cessie [37] proposed a global shrinkage method where regression estimates derived from least-squares or maximum likelihood were shrunk towards zero by a constant factor *c*, estimated using leave-one-out cross-validation (LOOCV). In OLS model with the assumption of homo-skedasticity, the value for *c* can be estimated using the formula [37]

$$c=1-\frac{p\hat{\sigma}^{2}}{ESS}$$

where $ESS=\sum_{i=1}^{n} \left( \hat{y_{i}}-\bar{y} \right){}^{2}= \sum_{i=1}^{n} \hat{y}_{i}^{2}$ is the explained sum of squares, $\hat{\sigma}^{2}$ is the estimate of residual variance and *p* the number of covariates. The adjusted linear predictor is [3]

$$\hat{y}_{i}= c\hat{\beta}_{1}x_{i1}+\ldots+ c\hat{\beta}_{p}x_{ip}$$

It turned out that the shrunken estimates ($\hat{\beta}_{j}^{*}= c\hat{\beta}_{j}$) gave lower prediction mean squared errors than the unshrunken least-squares estimates in the new data [3, 37]. The global shrinkage method can be used in both full and selected models, and its shrinkage behavior is similar to that of the lasso because it shrinks small and large nonzero coefficients equally. LOOCV and 10-fold CV will be used to estimate shrinkage factors as discussed in subsection 4.11.4.

**4.11.2 Parameterwise shrinkage**

An extension of global shrinkage method in which each regression estimate from a selected model was shrunk differently (i.e., $\hat{\beta}_{j}^{*}= c_{j}\hat{\beta}_{j}$) was proposed by [38], the so-called parameterwise shrinkage factors (PWSF). Again, the shrinkage factors $c_{j}$ were estimated using LOOCV. The adjusted linear predictor is [3]

$$\hat{y}_{i}= c_{1}\hat{\beta}_{1}x_{i1}+\ldots+ c_{p}\hat{\beta}_{p}x_{ip}$$

According to [38], PWSF should not be used with the full model because the estimated shrinkage factors cannot handle noise covariates. When used in a full model, the shrinkage factors of noise covariates can be negative. The global shrinkage and PWSF methods were shown to improve the predictive accuracy of models derived from backward elimination [3]. It is well-known that variable selection yields regression coefficients that are biased in absolute terms and need shrinkage [5, 30]. However, the bias is more pronounced in weak effect variables and negligible in strong effect variables [36]. For this reason, the PWSF approach that shrinks individual regression coefficients differently, similar to NNG, had better performance than global shrinkage in the simulation study comparing the two approaches [3]. LOOCV and 10-fold CV will be used to estimate PWSF shrinkage factors for the full model as well as models selected by best subset selection and backward elimination as discussed in subsection 4.11.4.

**4.11.3 Breiman’s shrinkage**

Breiman [10] proposed a shrinkage method that does not zero coefficients due to the quadratic penalty term. Let $x_{ij}^{*}=\hat{\beta}_{j}^{OLS}x_{ij}$ where $\hat{\beta}_{j}^{OLS}$ is the OLS estimate obtained from the full OLS model. The shrinkage factors $c=\left( c_{1},\ldots,c_{p} \right)^{T}$ are obtained by minimizing the objective function

$$\hat{c} \left( \lambda\right)=\text{arg }\text{min}_{c}\frac{1}{2n} {\sum_{i=1}^{n} \left( y_{i}-\sum_{j=1}^{p} c_{j}{x^{*}}_{ij} \right)}^{2}+\lambda\sum_{j=1}^{p} c_{j}^{2}, c_{j}\geq0, \lambda\geq0$$

and the shrunken regression coefficient is given by $\hat{\beta}_{j}^{*}\left( \lambda\right)=\hat{c}_{j}\hat{\beta}_{j}^{OLS},$ *j = 1,…,p*. When used after model selection, we replace $x_{ij}^{*}$ by $x_{ij}^{**}=\hat{\beta}_{j}^{OLS^{*}}x_{ij}^{(sel)}$ where $x_{j}^{(sel)}$ is the *jth* selected covariate and ${\hat{\beta}_{j}^{OLS}}^{*}$ is the corresponding OLS estimate. The shrinkage factors $c=\left( c_{1},\ldots,c_{k} \right)^{T}$ are obtained by minimizing the objective function

$$\hat{c} (\lambda)=\text{arg }\text{min}_{c}\frac{1}{2n} {\sum_{i=1}^{n} \left( y_{i}-\sum_{j=1}^{k} c_{j}{x^{**}}_{ij} \right)}^{2}+\lambda^{*}\sum_{j=1}^{k} c_{j}^{2}, c_{j}\geq0, \lambda\geq0$$

where $k\leq p$ is the number of covariates selected by a model selection procedure. The shrunken regression coefficient is given by $\hat{\beta}_{j}^{**}(\lambda^{*})=\hat{c}_{j}{\hat{\beta}_{j}^{OLS},}^{*}$ *j = 1,…,k*. The tuning parameters $\lambda$ and $\lambda^{*}$ will be estimated using a 10-fold CV. Breiman’s method of estimating shrinkage factors will be applied to the full model, as well as models selected by best subset selection and backward elimination.

**4.11.4 Estimation of global and parameterwise shrinkage factors**

Leave-one-out cross-validation was proposed for the estimation of global and PWSF shrinkage factors by [37, 38]. This approach requires fitting a statistical model *n* times (where *n* is the number of observations), which can be computationally intractable in simulation studies, especially when *n* is extremely large and each individual model is slow to fit. In addition, each model is developed on an almost identical set of observations, implying that the resulting models are nearly identical. Both LOOCV and 10-fold CV will be used to ascertain whether they yield similar results. The latter requires fitting a statistical model only 10 times, making it more practical for large problem sizes, and at least the training models are somewhat different. Recall that LOOCV is a special case of k-fold CV when *k = n,* hence, the procedure for estimating PWSF via LOOCV described by [39] can easily be adapted for k-fold CV, hence no further details are provided. Finally, it is important to mention that before conducting variable selection and estimating shrinkage factors, the covariates must be standardized to zero mean and unit variance if the scale used to measure individual covariates is different [38].

## Performance measures

### 5.1 False positives and false negatives rates

For each scenario, we will report false positive rates (FPR) and false negative rates (FNR) for individual variables and overall false positive and false negative rates for a variable selection approach. A false positive (FP) occurs for a variable $x_{j}$ when it is selected but its true regression coefficient is $\beta_{j}=0$. Then the FP rate for $\beta_{j}$ is the percentage of times an FP occurs for $\beta_{j}$ *i.e*. $FPR\left( \beta_{j} \right)=\left( \frac{1}{N}\sum_{i=1}^{N} 1\left( \hat{\beta}_{ij}\neq0 \right) \right)\times100,$where N is the number of simulation runs. The overall FPR of an approach is the average of the FP rates across all zero coefficients of β *i.e*., $\frac{1}{K}\sum_{j=1}^{K} FPR(\beta_{j})$ where K is the number of zero components.

A false negative (FN) is said to occur for a variable $x_{j}$when its true regression coefficient is $\beta_{j}\neq0$ but the variable is not selected. Then the FN rate for $\beta_{j}$ is the percentage of times FN occurs for $\beta_{j},$ *i.e*., $FNR\left( \beta_{j} \right)=\left( \frac{1}{N}\sum_{i=1}^{N} 1\left( \hat{\beta}_{ij}=0 \right) \right)\times100$, and the overall FNR is the average of the FN rates across all nonzero coefficients of $\beta_{j},$ *i.e*., $\frac{1}{M}\sum_{j=1}^{M} FNR(\beta_{j})$ where M is the number of nonzero coefficients. Graphical representations will be used to compare the overall FPR and FNR for all approaches. For example, a plot of FNR against SNR may allow us to uncover the relationship between FNR and SNR, and ease the comparison of different approaches.

**5.2 Model error**

Let $\boldsymbol{X}\in\mathbb{R}^{n\times p}$ be a matrix of covariates and $Y\in\mathbb{R}^{n}$ a vector of the response variable in the training dataset where$(x_{i}, y_{i})$, $i=1,\ldots,n$ are assumed to be independent and identically distributed (iid) random variables drawn from a distribution of$(\boldsymbol{X}, Y)$. Similarly, let $\boldsymbol{X}^{test}\in\mathbb{R}^{n\times p}$and $Y^{test}\in\mathbb{R}^{n}$ be a matrix of covariates and a vector of response in the test dataset respectively. Here, $(x_{i}^{test},y_{i}^{test})$ are again assumed to be iid randomly sampled from the same distribution of$\left( \boldsymbol{X},Y \right)$ but they are independent of $\left( x_{i}, y_{i} \right).$ Further, let $\hat{\beta}$ be estimated coefficients from one of the variable selection procedures such as the lasso and assume that the random error $\epsilon$is independent of $\boldsymbol{X}$ such that $E\left( \epsilon| \boldsymbol{X} \right)=0$ and$E(\epsilon\epsilon^{T}|\boldsymbol{X})= \sigma^{2}I_{n}$. Then, the mean-squared prediction error$(MSPE$) can be decomposed into two parts: $E\left[ \left( Y^{test}-{\boldsymbol{X}^{test}}^{T}\hat{\beta} \right)^{2} \right]=E\left[ \left( {\boldsymbol{X}^{test}}^{T}\hat{\beta}-{\boldsymbol{X}^{test}}^{T}\beta\right)^{2} \right]+\sigma^{2}$ where the expectation is taken over$(\boldsymbol{X}^{test}, Y^{test})$. The first component is the prediction error arising due to a lack of fit to the underlying model; often called ***model error*** (ME). The second component ($\sigma^{2}$) is the prediction error due to the noise (for details see [10, 22]). Since the second component is irreducible, we prefer to work directly with the first component (ME). We refer to [9] section 2.1.1 for more information about ME and irreducible error.

**Further analysis of ME using linear mixed model**

To assess how the simulation parameters affect the performance of ME and investigate the variability of ME within and between scenarios, we will fit a linear mixed model for each approach. This is because the analysis must account for the fact that responses from the same scenario are highly likely to be correlated (*i.e*., to yield a similar outcome). In repeated measures terminology, a scenario is equivalent to a “subject” while a simulation repetition is equivalent to an “occasion”. In total, we have 128 scenarios and 2,000 simulation repetitions. Each outcome of a scenario is measured J times (J = 2,000). Let $y_{ij}$ be the logarithm transformation of ME for the $ith$ scenario in the $jth$ simulation run. This implies that each scenario has a vector of J measurements $Y_{i}=\left( y_{i1},\ldots,y_{iJ} \right)^{T}$. Let $x_{ij}=\left( x_{ij1},\ldots,x_{ijp} \right)^{T}$ denotea design vector of the simulation parameters associated with $y_{ij}$, $i=1,\ldots128$, j = 1,…,2000. Then a linear mixed model is given by:

$$y_{ij}=\beta_{0}+b_{i}+\beta_{1}x_{ij1}+\beta_{2}x_{ij2}+\beta_{3}x_{ij3}+\beta_{4}x_{ij4}+\beta_{5}x_{ij5}+\beta_{6}x_{ij6}+\beta_{7}x_{ij7}+\beta_{8}x_{ij8}+\beta_{9}x_{ij9}+\beta_{10}x_{ij10}+\epsilon_{ij}$$

Where $x_{ij1}{-x}_{ij3}$ are dummy variables for configuration of $\beta$, $x_{ij4}{-x}_{ij6}$ are dummy variables for correlation structure (C), $x_{ij7}{-x}_{ij9}$ are dummy variables for *R^2^* and $x_{ij10}$is a dummy variable for sample size (n). $b_{i}$ is a scenario-specific random effect assumed to be sampled from a normal distribution with mean 0 and variance $\sigma_{b}^{2}$, *i.e.* $b_{i}\sim N\left( 0,\sigma_{b}^{2} \right)$and $\epsilon_{ij}\sim N(0, \sigma_{e}^{2})$. The boxplots of the random effects will help us to identify outlying scenarios [40, 41]. We will investigate whether adding two-way interaction terms to the linear predictor improves the model fit. In total, we have 45 two-way interaction terms and we will use a forward stepwise approach with a low nominal significance level of 1% for inclusion. The main aim is to identify stronger interactions and avoid adding (too) many false positive interactions. To get the big picture right instead of optimizing specific aspects, it is less critical if weaker interactions will not be identified with this strategy [42].

# Software implementations

### 6.1 Penalized methods

All simulations will be conducted using R software version 4.2.0 [43] and the R code for reproducing all the results will be made available at https://github.com/EdwinKipruto/simulation-study. Several R packages including glmnet [13] and penalized [44] implement the lasso. We will use the former for computational efficiency. The algorithm that solves the lasso problem will be used to obtain relaxed lasso and adaptive lasso solutions. The computational details for the latter are explained in section 3.5 of [12]. The nonnegative garrote solution hitherto is computed using the standard quadratic programming technique as demonstrated by [10] but can be computationally demanding for large-scale problems. However, it has been shown that the nonnegative garrote solution path can be solved using a modified LARS algorithm [45] with non-negativity constraints [11]. Thus, we will use a custom-made script that uses glmnet to obtain the shrinkage factors for the nonnegative garrote by constraining the lower bound of parameters to zero, thus yielding an efficient computational cost. Again, glmnet will be used to obtain ridge regression estimates that are required while constructing adaptive lasso and nonnegative garrote weights.

### 6.2 Classical and post-estimation shrinkage methods

The available R package that implements subset selection with impressive speed is known as leaps [34]. It uses a pure branch-and-bound algorithm of [31]. Thus, the best subset selection problems will be solved using the regsubset function in the leaps package which performs an exhaustive search and returns the best model for each size, where ‘best model’ is a model with the smallest residual sum of squares for each model size [9]. To select a single best model, AIC, BIC and CV will be used. BIC is automatically implemented in the function, while AIC and CV are not. As such, we will use a custom-made script to implement AIC and 10-fold CV as explained in [9]. The leaps package also implements backward elimination, hence the regsubset function will be used. To estimate global and parameterwise shrinkage factors using LOOCV and k-fold CV approach, we will use a custom-made script. Breiman's method of estimating post-estimation shrinkage factors will be implemented using a custom-made script. Lastly, we will simulate covariate variables $(X, X^{test})$ and random errors ($\epsilon$) using *rnorm* function in the stats package.

### 6.3 Fitting linear mixed models for ME

To estimate regression parameters of linear mixed models, the *lmer* function in the R package lme4 [46] will be used. This package has more efficient linear algebra tools, thus suitable for handling large computation problems. Also, it provides summary statistics for assessing the goodness of fit for models.

### Random number seed

Researchers across a wide range of scientific disciplines have been advocating for reproducible research. To ease the reproducibility of simulation studies, a random-number seed must be provided. Different seeds produce different results where a seed is defined as an initial value used by random number functions to generate a sequence of random numbers. If different seeds are used to generate pseudorandom numbers, the seeds must not follow a pattern because the pseudorandom numbers you get are nothing more than the seeds you ran through a mathematical function. Unless the seeds you choose are also random, the results you get will not be random. Thus, the use of dates or times of the day is not recommended [47]. In a simulation study, the seed must be set only once at the start of the simulation, and any number can be used as a seed [47, 48]. We will use a seed of **472095** with Mersenne-Twister (MT) as the random number generator (RNG), which is the default in R software. MT is well-known for producing good random numbers for practical pseudorandom number generators, which are essential in simulation studies [49].

- - - 1. References

1. Hastie T, Tibshirani R, Tibshirani R. Best subset, forward stepwise or lasso? Analysis and recommendations based on extensive comparisons. Stat Sci. 2020;35(4):579–92.
2. Bertsimas D, King A, Mazumder R. Best subset selection via a modern optimization lens. Ann Stat. 2016;44(2):813–52.
3. van Houwelingen HC, Sauerbrei W. Cross-validation, shrinkage and variable selection in linear regression revisited. Open J Stat. 2013;03(02):79–102.
4. Franklin JM, Schneeweiss S, Polinski JM, Rassen JA. Plasmode simulation for the evaluation of pharmacoepidemiologic methods in complex healthcare databases. Comput Stat Data Anal. 2014;72:219–26.
5. Harrell FE. Regression modeling strategies: With applications to linear models, logistic and ordinal regression, and survival analysis. Cham, Switzerland: Springer International Publishing; 2016;68-73,112,209.
6. Tibshirani, R. Regression shrinkage and selection via the lasso. *Journal of the Royal Statistical Society: Series B (Methodological)*, 1996;58(1):267-88.
7. Breiman L. Heuristics of instability and stabilization in model selection. Ann Stat. 1996;24(6):2350–83.
8. Hastie T, Tibshirani R, Kondofersky I, Wainwright M. Statistical learning with sparsity: The lasso and generalizations. Boca Raton: CRC press. Biometrics. 2018;74(2):769–769.
9. James G, Witten D, Hastie T, Tibshirani R. An introduction to statistical learning: With applications in R. 1st ed. New York, NY: Springer; 2013.
10. Breiman L. Better subset regression using the nonnegative garrote. Technometrics. 1995;37(4):373–84.
11. Yuan M, Lin Y. On the non-negative garrotte estimator. J R Stat Soc Series B Stat Methodol. 2007;69(2):143–61.
12. Zou H. The adaptive lasso and its oracle properties. J Am Stat Assoc. 2006;101(476):1418–29.
13. Friedman J, Hastie T, Tibshirani R. Regularization paths for generalized linear models via coordinate descent. J Stat Softw. 2010;33(1):1–22.
14. Zhao P, Yu B. On model selection consistency of Lasso. J Mach Learn Res. 2006;7:2541-63.
15. Buehlmann P, van de Geer S. Statistics for high-dimensional data: Methods, theory and applications. 2011th ed. Berlin, Germany: Springer; 2013.
16. Tibshirani R, Wasserman L. Sparsity, the lasso, and friends. Lecture notes from “Statistical Machine Learning,” Carnegie Mellon University, Spring. 2017.
17. Huang J, Ma S. and Zhang C.H. Adaptive Lasso for sparse high-dimensional regression models. *Statistica Sinica*. 2018;18(4):1603-1618.
18. Zou H, Hastie T. Regularization and variable selection via the elastic net. J R Stat Soc Series B Stat Methodol. 2005;67(2):301–20.
19. Meinshausen N. Relaxed lasso. Comput Stat Data Anal. 2007;52(1):374–93.
20. Benner A, Zucknick M, Hielscher T, Ittrich C, Mansmann U. High-dimensional Cox models: the choice of penalty as part of the model building process. Biom J. 2010;52(1):50–69.
21. Fan J, Li R. Variable selection via nonconcave penalized likelihood and its oracle properties. J Am Stat Assoc. 2001;96(456):1348–60.
22. Hastie T, Tibshirani R, Friedman JH. The elements of statistical learning: Data mining, inference, and prediction. 2nd ed. New York, NY: Springer; 2009.
23. Friedman J, Hastie T, Tibshirani R. Regularization paths for generalized linear models via coordinate descent. J Stat Softw. 2010; 33(1):1–22.
24. Zou H, Hastie T, Tibshirani R. On the “degrees of freedom” of the lasso. Ann Stat. 2007;35(5):2173–92.
25. Yang Y. Can the strengths of AIC and BIC be shared? A conflict between model indentification and regression estimation. Biometrika. 2005;92(4):937–50.
26. Burnham KP, Anderson DR. Multimodel inference: Understanding AIC and BIC in model selection. Sociol Methods Res. 2004;33(2):261–304.
27. Wang H, Li R, Tsai C-L. Tuning parameter selectors for the smoothly clipped absolute deviation method. Biometrika. 2007;94(3):553–68.
28. Fan Y, Tang CY. Tuning parameter selection in high dimensional penalized likelihood. J R Stat Soc Series B Stat Methodol. 2013;75(3):531–52.
29. Luo S, Chen Z. Extended BIC for linear regression models with diverging number of relevant features and high or ultra-high feature spaces. J Stat Plan Inference. 2013;143(3):494–504.
30. Miller AJ. Subset selection in regression. Monographs on statistics and applied probability, volume 95. London, England: CRC Press; 2002.
31. Furnival GM, Wilson RW. Regressions by leaps and bounds. Technometrics. 2000;42(1):69–79.
32. Sauerbrei W, Perperoglou A, Schmid, M., Abrahamowicz M, Becher H, Binder H, Dunkler D, Harrell F.E, Royston P, Heinze G, for TG2 of the STRATOS initiative. State of the art in selection of variables and functional forms in multivariable analysis - outstanding issues. *Diagnostic and Prognostic research*. 2020;4(1), 1-18.
33. Mantel N. Why stepdown procedures in variable selection. Technometrics. 1970;12(3):621.
34. Lumley T. based on Fortran code by Alan Miller. leaps: Regression Sub- set Selection. R package version 3.1. 2020. <https://CRAN.R-project.org/package=leaps>
35. Heinze G, Wallisch C, Dunkler D. Variable selection - A review and recommendations for the practicing statistician. Biom J. 2018;60(3):431–49.
36. Royston P, Sauerbrei W. Multivariable model - building: A pragmatic approach to regression anaylsis based on fractional polynomials for modeling continuous variables. Wiley series in probability and statistics. John Wiley & Sons; 2008.
37. Van Houwelingen JC, Le Cessie S. Predictive value of statistical models. Stat Med. 1990;9(11):1303–25.
38. Sauerbrei W. The use of resampling methods to simplify regression models in medical statistics. J R Stat Soc Ser C Appl Stat. 1999;48(3):313–29.
39. Dunkler D, Sauerbrei W, Heinze G. Global, parameterwise and joint shrinkage factor estimation. J Stat Softw. 2016;69(8):1-19.
40. Aerts M, Molenberghs G, Ryan LM, Geys H, editors. Topics in modeling of clustered data. London, England: CRC Press; 2019.
41. Molenberghs G, Verbeke G. Models for Discrete Longitudinal Data. New York, NY: Springer; 2005.
42. Sauerbrei W, Royston P, Binder H. Selection of important variables and determination of functional form for continuous predictors in multivariable model building: selection of variables and functional forms. Stat Med. 2007;26(30):5512–28.
43. R Core Team. R: A language and environment for statistical computing. R Foundation for StatisticalComputing, Vienna, Austria. 2022. URL: <https://www.R-project.org/>.
44. Goeman J, Meijer R, Chaturvedi N, Lueder M, Goeman M.J, Rcpp I and Rcpp L. Package ‘penalized’. R package version. 2018.
45. Efron B, Hastie T, Johnstone I, Tibshirani R. Least angle regression. Ann Stat. 2004;32(2):407–99.
46. Bates D, Mächler M, Bolker B, Walker S. Fitting Linear Mixed-Effects Models using lme4. arXiv [stat.CO]. 2014.
47. StataCorp LP. Stata base reference manual. College Station, Texas: Stata. 2005.
48. Morris TP, White IR, Crowther MJ. Using simulation studies to evaluate statistical methods: Using simulation studies to evaluate statistical methods. Stat Med. 2019;38(11):2074–102.
49. Matsumoto M, Nishimura T. Mersenne twister: A 623-dimensionally equidistributed uniform pseudo-random number generator. ACM Trans Model Comput Simul. 1998;8(1):3–30.
